# *In vitro* and *in vivo* antitumour effects of phenylboronic acid against mouse mammary adenocarcinoma 4T1 and squamous carcinoma SCCVII cells

**DOI:** 10.1080/14756366.2017.1384823

**Published:** 2017-10-26

**Authors:** Maja Marasovic, Sinisa Ivankovic, Ranko Stojkovic, Damir Djermic, Borivoj Galic, Mladen Milos

**Affiliations:** a Faculty of Chemistry and Technology, University of Split, Split, Croatia;; b Division of Molecular Medicine, Rudjer Boskovic Institute, Zagreb, Croatia;; c Division of Molecular Biology, Rudjer Boskovic Institute, Zagreb, Croatia;; d Faculty of Science, University of Sarajevo, Sarajevo, Bosnia and Herzegovina

**Keywords:** Boron, phenylboronic acid, antitumour, 4T1 cells, SCCVII cells

## Abstract

The cytotoxic activity of phenylboroxine acid was evaluated *in vitro* on mouse mammary adenocarcinoma 4T1, mouse squamous cell carcinoma SCCVII, hamster lung fibroblast V79 and mouse dermal fibroblasts L929 cell lines. The cytotoxic effects were dose dependent for all tested tumour and non-tumour cell lines. Under *in vivo* conditions, three application routes of phenylboronic acid were studied: intra-peritoneal (i.p.), intra-tumour (i.t.) and per-oral. After tumour transplantation in syngeneic mice, phenylboronic acid was shown to slow the growth of both tumour cell lines (4T1 and SCCVII) compared with the control. The inhibitory effects were pronounced during the application of phenylboronic acid. For both tested tumour cell lines, the most prominent antitumour effect was obtained by intraperitoneal administration, followed significantly by oral administration.

## Introduction

Generally, the majority of current cancer treatments control the rapid proliferation of primary tumour cells. However, conventional therapeutics are not sufficiently selective for tumourigenic cells. Damage to normal tissues could be significantly reduced if a potential therapeutic compound selectively impacts tumour cells while avoiding normal, healthy cells^1^. Therefore, it is very important to identify highly selective and non-toxic compounds. Baker et al.^2^ described the therapeutic potential of boron-containing compounds and suggested their use as a new class of anticancer agents. Other medicinal chemistry studies have resulted in the commercialisation of peptidylboronic acid antineoplastic drugs, such as Velcade or Bortezomid^3^^,^^4^. In the development of boron-based enzyme inhibitors as potential antitumour drugs, increased targeting specificity limits their side effects^5^. Epidemiological, *in vitro*, and animal studies have revealed possible roles for boric acid as a chemotherapeutic agent^6–8^. Boric acid is a mild organic Lewis acid with structural features similar to carbon, allowing it to act as a competitive inhibitor for many carbon-containing substrates. A few studies^9^^,^^10^ have indicated phenylboronic acid (PBA) as being more potent than boric acid in targeting metastatic and proliferative properties of cancer cells. These studies have shown that PBA can selectively inhibit human prostate and breast cancer cell migration and decrease cancer cell viability. In recent studies^11^^,^^12^, *in vivo* antitumour examinations demonstrated that PBA-enriched nanoparticles have superior efficacy in restricting tumour growth and prolonging the survival time of tumour-bearing mice than free drugs.

These aforementioned studies have indicated the promising direction of studying the effects of PBA inhibition properties on other types of cancer cell lines. Therefore, as part of our project, “Research on antitumour properties of different derivatives of boronic acid”, we decided to investigate *in vitro* and *in vivo* antitumour effects of PBA against mouse mammary adenocarcinoma 4T1 and squamous carcinoma SCCVII cells. *In vivo* experiments were performed on mice using three modes of administration: intra-peritoneal, intra-tumour and per-oral.

## Materials and methods

### Tested compound and cell lines

PBA solutions (Sigma-Aldrich, Buchs, Switzerland) were prepared by dissolution in phosphate buffer (Fisher Chemical, Wien, Austria). Mouse mammary adenocarcinoma 4T1 cell line was purchased from American Type Culture Collection (ATCC, Manassas, VA) and mouse squamous cell carcinoma SCCVII cell line was obtained from BC Cancer Research Centre (Vancouver, Canada). Both SCCVII and 4T1 murine tumour cells are widely accepted animal models for the investigation of these types of tumours in humans: SCCVII for human head and neck tumours^13^ and 4T1 for human breast adenocarcinomas^14^. The syngeneic transplantation model allowed us to test the toxicity of PBA on the same cells *in vivo* and *in vitro*. Cells were grown in RPMI 1640 medium (Sigma-Aldrich, Buchs, Switzerland) supplemented with 10% foetal calf serum (FCS; Sigma-Aldrich, Buchs, Switzerland), in a humidified 5% CO_2_, 37 °C atmosphere.

### Animals and tumour models

The mice used in the study were obtained from Ruđer Boškovic Institute’s breeding colony. Two mouse strains, BALB/c syngeneic with 4T1 cells and C3H/H syngeneic with SCCVII cells, were used. The animals were ∼3-months-old and weighed 20–23 g. They were provided standard diet (Mucedola S.R.L., Milan, Italy) and tap water *ad libitum*. The animals were kept under conventional conditions: 12/12 h light/dark rhythms, 22 °C temperature and 55% humidity. Animals were treated according to the Animal Welfare Regulations. Each experimental group consisted of seven animals. Mouse tumour models of mammary adenocarcinoma 4T1 and squamous cell carcinoma SCCVII were established by injecting 5 × 10^5^ tumour cells subcutaneously into the right thighs of syngeneic mice.

All experimental procedures were performed in accordance with the current guidelines for the care of laboratory animals (including the use of the 3Rs procedures). The experiments were terminated before the mean tumour diameter exceeded 15 mm as recommended in the 2010 guidelines for the welfare and use of Animals in cancer research^15^.

### Determination of cytotoxic activity *in vitro*

For evaluation of cell viability and cytotoxic effects of PBA on cultured cells, a crystal violet staining assays was used^16^. This test only determines living cells at the time of fixation and staining of the examined samples, while the dead cells are removed by washing of cell cultures. Experiments were carried out in 96-well microtitre plates; 1 × 10^4^ tumour cells/250 μl medium were added to each well. After 24 h, when the cells reached confluence, old cultured media were replaced with fresh media. The tested compound PBA was added to the cultures to final concentrations of 0.1, 1.0 and 10.0 mg/ml (0.82, 8.2 and 82.0 mM). Control cells were incubated in RPMI medium without the tested substance. The cells were incubated for an additional 24 h. Cytotoxicity tests with crystal violet were performed to measure cell growth inhibition rates. Briefly, cells were fixed by the addition of a 3% solution of formalin for 15 min, washed with deionised water and dried in air. Afterwards, cells were stained with 0.1% crystal violet for 20 min, then extensively washed with deionised water and left to dry overnight. The dye was extracted from the cells using a 10% solution of acetic acid. Then, absorbance was measured using a microplate reader. The absorbance at 590 nm is proportional to the number of surviving cells. Each experiment was performed in quadruplicate. Inhibition of cell growth relative to controls was calculated according to the formula: inhibition of cell growth I% = (C − T)/C × 100, where T denotes the mean absorbance of treated cells and C indicates the mean absorbance of untreated (control) cells. The LC_50_ concentrations were estimated using probit analysis^17^^,^^18^.

### Tumour treatment and evaluation of antitumour activity *in vivo*

The treatment was started on Day 9 after transplantation, when tumour volumes reached ∼250–300 mm^3^. For both experiments (cell lines 4T1 and SCCVII), mice were randomly divided into four groups (seven mice per group). Three application routes of PBA were studied: intra-peritoneal (i.p.), intra-tumour (i.t.) and per-oral. Briefly, 300 μl saline with a PBA dose of 100 mg/kg of mice (average body weight of 20–23 g) were administered once a day for nine consecutive days starting from Day 10. The total received dose was 900 mg/kg of mice for each mode of administration. Control mice received the same volumes of saline without PBA. The dosage regimen applied in this study was well-tolerated by the animals with no signs of overtoxicity, i.e. the animals were generally in good condition and did not show significant weight loss. Available literature has also suggested low toxicity of boron-containing acids, including PBA. Thus, in mice, a high LD_50_ dose of 900 mg/kg for acute toxicity was determined^19^. The dose used in our study (100 mg/kg) was nine time lower than the LD_50_ dose.

Tumour growth was observed by measuring three orthogonal tumour diameters (A, B, and C) with a calliper. Tumour volume was calculated by the formula V = ABCπ/6. The inhibition of tumour growth was calculated by the formula: TGI (%) = (C−T)/C × 100, where C is the mean tumour volume of the control (untreated) group, and T is the mean tumour volume of the treated groups. Significant differences in tumour volumes between the groups were determined by one-way analysis of variance (ANOVA) and Tukey’s *post hoc* test of multiple comparisons. *p* ≤ .05 was considered significant.

## Results

### Cytotoxic activity *in vitro*

**Figure 1.**
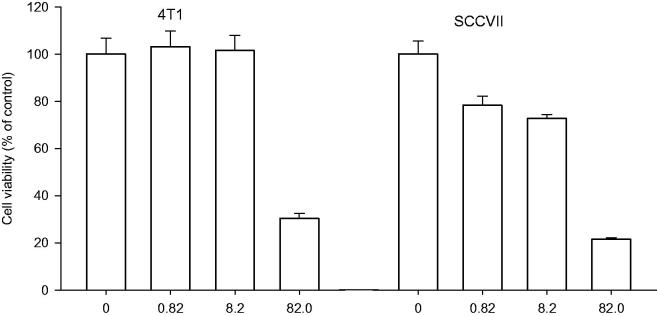
Cytotoxic effect of PBA 0.1, 1.0 and 10 mg/ml (0.82, 8.2 and 82 mM) on mammary adenocarcinoma 4T1 and squamous cell carcinoma SCCVII. Cells survival rate measured by crystal violet assay. Absorbance at 590 nm is proportional to the number of surviving cells. Each experiment was done in quadruplicate. Inhibition of cell growth I (%) relative to controls was calculated according to the formula: I = (C − T)/C × 100, where T denotes the mean absorbance of treated cells, and C indicates the mean absorbance of untreated cells, without the addition of PBA.

**Figure 2.**
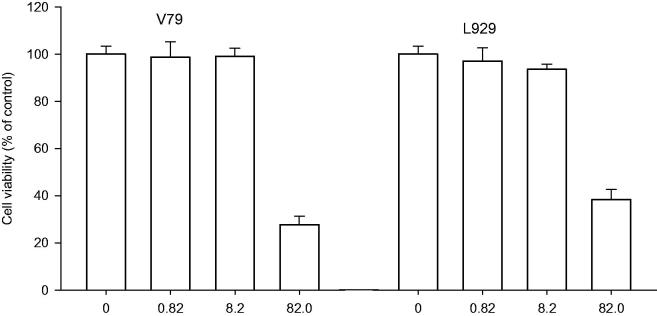
Cytotoxic effect of PBA 0.1, 1.0 and 10 mg/ml (0.82, 8.2 and 82 mM) on hamster lung fibroblast V79 and mouse dermal fibroblasts L929 cell lines. Cells survival rate measured by crystal violet assay. Absorbance at 590 nm is proportional to the number of surviving cells. Each experiment was done in quadruplicate. Inhibition of cell growth I (%) relative to controls was calculated according to the formula: I = (C − T)/C × 100, where T denotes the mean absorbance of treated cells, and C indicates the mean absorbance of untreated cells, without the addition of PBA.

The cytotoxic activities of PBA at different concentrations 0.1, 1.0 and 10 mg/ml (0.82, 8.2 and 82 mM) were evaluated on mouse mammary adenocarcinoma 4T1 and squamous cell carcinoma SCCVII cell lines (Figure 1) and on hamster lung fibroblast V79 and mouse dermal fibroblasts L929 cell lines (Figure 2). Generally, the cytotoxic effect of PBA was dose-dependent. In all tested tumour and non-tumour cell lines, 10 mg/ml PBA significantly reduced the number of surviving cells compared with the control group. However, some differences in the sensitivity of cells were observed at 0.1 and 1.0 mg/ml PBA. These concentrations did not significantly affect the growth of the 4T1, V79 and L929 cells; however, 22.0 and 28.1% inhibition were observed for the SCCVII cells. The LC_50_ values were estimated to be 4.5 and 7.5 mg/ml for the SCCVII and 4T1 cells and 6.0 and 7.5 mg/ml for the L929 and V79 cells, respectively.

### Antitumour activity *in vivo*

**Figure 3.**
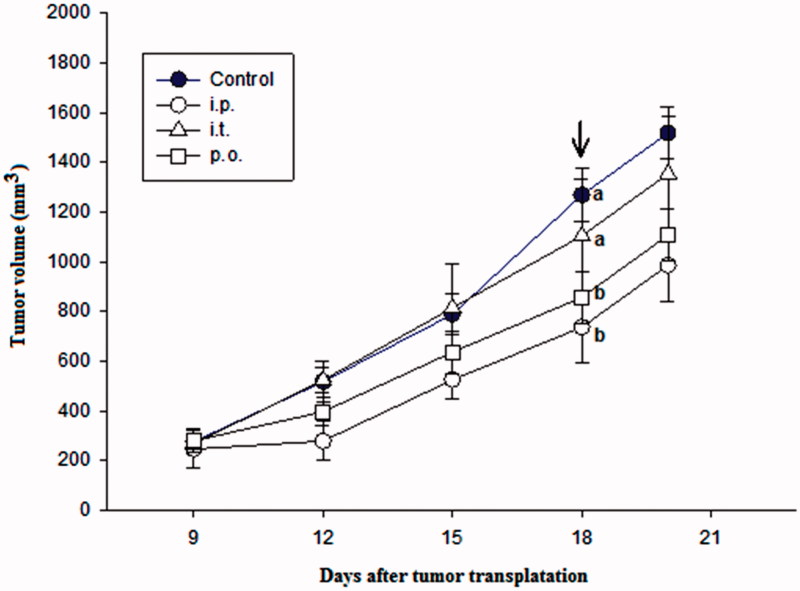
The effect of intra-peritoneal (i.p.), intra-tumour (i.t.) and per-oral (p.o.) application of PBA on the growth of squamous cell carcinoma SCCVII transplanted into mouse thigh. PBA was injected in a dose of 100 mg/kg once a day for nine consecutive days starting from Day 9 after tumour transplantation. Each experimental group consisted of seven animals. Different letters beside the symbols indicate significant differences between the groups (*p*<.05, Tukey’s *post hoc* test) at the end of the treatment (arrow).

Antitumour activity *in vivo* was assessed on the same tumour models as the *in vitro* experiments. Preliminary experiments showed that the intra-peritoneal application of 50 mg/kg of PBA in a single dose was well-tolerated by all mice. The same results were achieved when PBA was applied as a single 50 mg/kg intra-tumoural dose. When PBA was administered per-orally in five consecutive days in five doses of 50 mg/kg, PBA was also well-tolerated by all mice. The effects of intra-peritoneal (i.p.), intra-tumour (i.t.) and per-oral (p.o.) applications of PBA on the growth of squamous cell carcinoma SCCVII is presented in Figure 3. The intraperitoneal and oral applications significantly slowed tumour growth compared with control, whereas no significant delay in tumour growth was observed when PBA was administered intratumourally.

**Figure 4.**
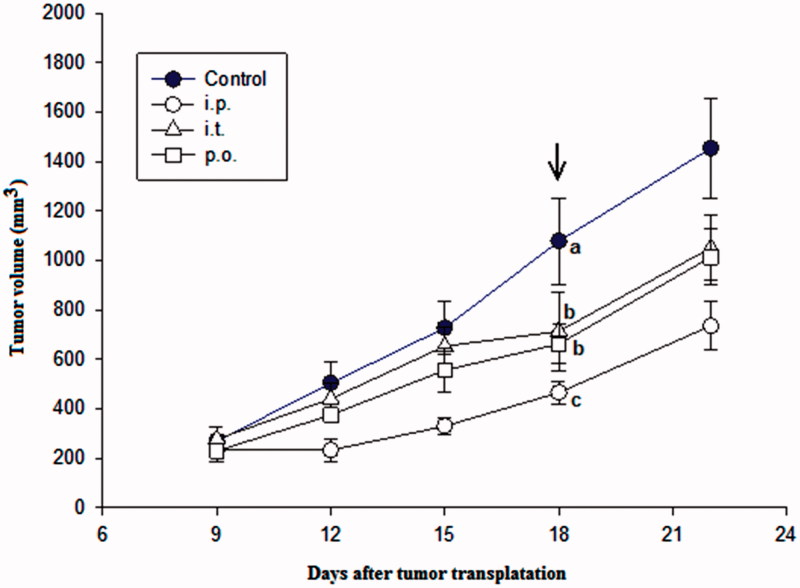
The effect of intra-peritoneal (i.p.), intra-tumour (i.t.) and per-oral (p.o.) application of PBA on the growth of mammary adenocarcinoma 4T1 transplanted into mouse thigh. PBA was injected in a dose of 100 mg/kg once a day for nine consecutive days starting from Day 9 after tumour transplantation. Each experimental group consisted of seven animals. Different letters beside the symbols indicate significant differences between the groups (*p* < .05, Tukey’s *post hoc* test) at the end of the treatment (arrow).

PBA significantly reduced the growth of adenocarcinoma 4T1 in all three modes of administration (Figure 4). The intra-peritoneal application was significantly more effective than the intra-tumour or per-oral applications with a tumour growth inhibition of ∼57% compared with control at the end of treatment (Figure 4). Table 1 shows different measured tumour parameters after transplantation of mammary adenocarcinoma 4T1 and squamous carcinoma SCCVII cells in syngeneic mice and after treatment by PBA with the different administration routes. All parameters clearly show that PBA has antitumour activity in both cell lines, especially when administered intra-peritoneally. The difference in volumes for treated versus control 4T1 tumour after the ninth day of the i.p. treatment was 613 mm^3^, which represents a 57% tumour growth inhibition and 7 days tumour growth delay.

**Table 1.** Tumour parameters after transplantation of mammary adenocarcinoma 4T1 and squamous carcinoma SCCVII cells in syngeneic mouse and their treatment by PBA in the different administration routes.

Cells	Mode	T_9_–C_9_ (mm^3^)	TGI_9_ (%)	TGD_700_ (days)
4T1	i.p.	613	57	7
	i.t.	365	34	4
	p.o.	417	39	4
SCVII	i.p.	532	42	4
	i.t.	164	13	0
	p.o.	411	32	2.5

T_9_–C_9_: the difference in volumes for treated versus control tumour after ninth day of treatment.

TGI_9_%: tumour growth inhibition; TGI% = (1 − T/C) × 100; where, C is the mean tumour volume of control group and T is the mean tumour volume of treated groups after ninth day of treatment.

TGD_700_: tumour growth delay presents the difference in days for treated versus control tumour to reach a volume 700 mm^3^.

Mode: i.t. (intra-peritoneal), i.t. (intra-tumour), and p.o. (per-oral).

## Discussion

This is the first report showing that PBA can inhibit the growth of mouse mammary adenocarcinoma 4T1 and mouse squamous cell carcinoma SCCVII. The data presented indicated that PBA is capable of inhibiting growth in the selected cell lines *in vitro* and *in vivo* by three modes of administration, i.e. intra-peritoneal, intra-tumour and per-oral. As presented in the previous section, at PBA concentrations <1 mg/ml (8.2 mM), no inhibitions in tumour 4T1 cells *in vitro* were observed. Similar results were observed with non-tumour cells V79 and L929, whereas in tumour SCCVII cells, partial growth inhibition was seen. Significant growth inhibition LC_50_ values were estimated at 4.5–7.5 mg/ml demonstrating no significant differences in inhibition between tumour cells and non-tumour cells.

Apoptosis is one of the most potent defences against cancer. This process eliminates potentially deleterious mutated cells^20^ and enables the renewal of normal, healthy cells. The mechanism whereby PBA inhibits cell growth *in vitro* and *in vivo* remains unknown. The sialic acid is known to be over-expressed on the cell surface of tumour cells and the increasing expression of sialic acid was previously shown on 4T1 cells^21^ and on SCCVII cells^22^. Since it has been reported that PBA possess high affinity to the sialic acid receptors^23^, it suggests that PBA could affect tumour cells via sialic acid receptors.

Previously, PBA had been suggested to act as proteasome inhibitors. The boronic acid motif acts as a transition state analogue, forming hydrogen and covalent bonds in the enzyme active site. For example, the inhibition constants of chymotrypsin at pH 7 for simple PBA were found to be K_i_ = 1.96 × 10^−4^ M compared with K_i_ = 1.0 × 10^−2^ M of benzyl alcohol, which lacks the boronic acid motif. Evidence suggested that the inhibition activity was due solely to the boronic acid. Slight changes in pH can result in the release of the inhibitor from the active site, enabling the use of boronic acids as biological probes and in therapeutics^24^. Therefore, we compared *in vitro* and *in vivo* PBA antitumour activities using the same tumour cell lines. *In vivo* experiments were performed intra-peritoneally, intra-tumourally and per-orally. With respect to the mode of administration, the results observed were generally similar for both tumour models, i.e. the most pronounced effect was obtained by intraperitoneal administration followed by oral and intratumoural administrations. The remarkable inhibition of tumour growth after oral administration suggested that PBA is not metabolised after oral administration or that its metabolites have similar antitumour effects. These metabolites, such as arylboronic acids and their esters, could have selectively reacted with H_2_O_2_ and metabolised the formation of boronate intermediates that rapidly hydrolyse to release phenols, borate esters, and boric acids^25^. Boronic acids and esters do not appear to have intrinsic toxicity issues; the boric acid end product is considered non-toxic to humans^26^. However, different prodrugs coupled with an ROS trigger unit have been observed to be triggered by H_2_O_2_ to release active anticancer drugs (effectors) with selective toxicity towards cancer cells^27^^,^^28^. Therefore, boronic acid could be used to develop novel methods to release pharmacologically active species triggered by high levels of H_2_O_2_ found in cancer cells^29^. Such agents have the potential to kill malignant cells while leaving healthy cells relatively untouched. These boronic acids also provide an excellent opportunity to evaluate the feasibility of the ROS-activated prodrug approach.

Recently, halogenated boroxines, e.g. K_2_[B_3_O_3_F_4_OH], have been suggested for use in the prevention and/or treatment of benign or malignant changes in epidermal tissue^30^. This compound was listed as a promising new therapeutic for cancer diseases. Our previous study^31^ showed that this compound affected the *in vitro* and *in vivo* growth of 4T1 mammary adenocarcinoma, B16F10 melanoma and squamous cell carcinoma SCCVII cells. In previous studies, boronic acid derivative K_2_[B_3_O_3_F_4_OH] was hypothesised to reduce catalase^32^, carbonic anhydrase^33^ and horseradish peroxidase^34^ activity by increasing the concentration of H_2_O_2_ to produce beneficial effects in tumour tissues alone. Considering these results, the same mode of action for PBA could be suggested but would require further study.

## Conclusion

As outlined by Baker et al.^2^, boron compounds could be become widely used in future drug discovery research. Our work showed that PBA proved to be highly cytotoxic on tumour cells and, even after repeated administration in large doses, was well-tolerated by mice. Future work will focus on elucidating the antitumour mechanism of PBA.
